# Systematic screening of 96 *Schistosoma mansoni* cell-surface and secreted antigens does not identify any strongly protective vaccine candidates in a mouse model of infection

**DOI:** 10.12688/wellcomeopenres.15487.1

**Published:** 2019-10-16

**Authors:** Cecile Crosnier, Cordelia Brandt, Gabriel Rinaldi, Catherine McCarthy, Colin Barker, Simon Clare, Matthew Berriman, Gavin J. Wright

**Affiliations:** 1 Wellcome Sanger Institute, Cambridge, CB10 1SA, UK

**Keywords:** Schistosoma mansoni, schistosomiasis, vaccine, cell-surface antigens, antibody

## Abstract

**Background:** Schistosomiasis is a major parasitic disease affecting people living in tropical and sup-tropical areas. Transmission of the parasite has been reported in 78 countries, causing significant morbidity and around 200,000 deaths per year in endemic regions. The disease is currently managed by the mass-administration of praziquantel to populations at risk of infection; however, the reliance on a single drug raises the prospect of parasite resistance to the only treatment widely available. The development of an effective vaccine would be a more powerful method of control, but none currently exists and the identification of new immunogens that can elicit protective immune responses therefore remains a priority. Because of the complex nature of the parasite life cycle, identification of new vaccine candidates has mostly relied on the use of animal models and on a limited set of recombinant proteins.

**Methods:** In this study, we have established an infrastructure for testing a large number of vaccine candidates in mice and used it to screen 96 cell-surface and secreted recombinant proteins from *Schistosoma mansoni*. This approach, using standardised immunisation and percutaneous infection protocols, allowed us to compare an extensive set of antigens in a systematic manner.

**Results:** Although some vaccine candidates were associated with a statistically significant reduction in the number of eggs in the initial screens, these observations could not be repeated in subsequent challenges and none of the proteins studied were associated with a strongly protective effect against infection.

**Conclusions: **Although no antigens individually induced reproducible and strongly protective effects using our vaccination regime, we have established the experimental infrastructures to facilitate large-scale systematic subunit vaccine testing for schistosomiasis in a murine infection model.

## Introduction

With over 200 million individuals affected every year and a third of all countries considered endemic, schistosomiasis remains one of the most widespread parasitic diseases on the planet. It is one of the world’s major global health problems, being responsible for 200,000 deaths annually ^1, 2^. Infections in humans are caused by five main species of platyhelminth parasites of the genus *Schistosoma*, of which *S. mansoni* and *S. haematobium* are the most prevalent. Infection in humans begins with the penetration of the skin by free-swimming cercariae, which triggers their transformation into schistosomula. After a few days of maturation in the dermis, these enter the bloodstream and progressively develop into male and female worms that pair up in the blood vessels of their host’s liver. Pairs ultimately migrate towards their final place of residence: the mesenteric vessels of the bowel in the case of species causing intestinal schistosomiasis, or the venous plexus of the bladder for *Schistosoma haematobium,* where female worms start laying eggs four to six weeks after infection. Despite the widespread incidence and debilitating effects of schistosomiasis in chronically infected patients, praziquantel is the only drug routinely used to treat established infections in populations living in endemic areas. The incomplete efficacy of the treatment, its lower efficiency at killing immature schistosomula and the increasing risk of drug resistance ^3^ make the discovery of new therapeutics, and in particular vaccines, even more pressing ^4, 5^. Yet, despite many years of research, very few vaccine candidates have progressed to clinical trials ^6^. Two *S. mansoni* antigens (Sm-14, Sm-TSP-2) are being tested in humans, while a third antigen, Sm-p80, should soon enter phase I studies ^7^. In the case of *S. haematobium,* Sh28-GST has been the only clinically tested candidate against the urogenital form of the disease ^8^.

The publication of the parasite’s genome ^9^ and advances in transcriptional and proteomics studies ^10– 18^ have revealed many potential candidates whose protective effects are yet to be tested in animal models. Proteins secreted by the parasite or present on its surface are generally considered good vaccine candidates because of their direct accessibility to vaccine-induced antibodies. The surface of the adult schistosome, however, is covered by a thick tegumental double phospholipid bilayer shielding the parasite from host antibodies and capturing host antigens ^19, 20^, allowing it to survive for many years, sometimes decades, in the vasculature of infected individuals ^10^. Targeting the parasite at a different stage of its life cycle, when it could be more vulnerable to the host humoral immune system, has been proposed as a possible approach ^21^.

Immediately after penetrating the skin, the early schistosomula undergo an extensive surface remodelling, leaving their external surface free of the dense glycocalyx that was covering them at the cercarial stage ^22^. In support of this idea, infection of various animal models with irradiated cercariae can elicit immunological protection to reinfection ^23, 24^, suggesting that the parasite is more susceptible to killing by the host in the first few days that follow infection. The use of attenuated parasites as a vaccine is, however, not suitable both in terms of practicability (they can be difficult to prepare and may suffer from batch-to-batch variations) and safety (each batch needs to be thoroughly tested to guarantee the efficacy of attenuation). For these reasons, modern vaccines are often based on chemically defined recombinant proteins, which are much easier to manufacture. Nevertheless, for these proteins to work efficiently as vaccines, they must be in a conformation that will elicit the generation of host antibodies that recognise antigens natively expressed on the surface of the parasite. Although popular heterologous expression systems such as bacteria and yeast have been successfully used for the generation of intracellular proteins, they are not generally regarded as suitable for the systematic production of secreted and cell-surface proteins, which require the formation of disulphide bonds for their correct folding and the addition of glycan structures. Also, the differences in adjuvants, routes of delivery, immunisation regimes and heterologous expression systems used to produce the antigens can lead to contradictory results on the protective effect of certain antigens ^25– 27^. We have recently described a library of secreted and cell-surface *S. mansoni* proteins whose selection was based on published proteomics studies and transcriptional data at the schistosomula stage ^28^. Importantly, these proteins were expressed in human embryonic kidney (HEK)-293 cells, to increase the chances of forming disulphide bonds that are critical for the correct tertiary folding of extracellular proteins. Testing against sera from patients living in a high-endemicity area showed that most of them contain heat-labile conformational epitopes ^28^.

In this study, we have produced and purified 96 antigens from this library for immunisation in mice. By establishing infrastructures for large-scale systematic challenges and standardised infection parameters, we have systematically tested the protective efficacy of these antigens in immunised mice against infection with *S. mansoni* cercariae.

## Methods

### Expression and purification of recombinant antigens from mammalian HEK-293-E cells

Antigens ( Table 1) were produced by transient transfection of human embryonic kidney (HEK)-293-E or HEK-293-6E cells (provided by Yves Durocher, NRC, Montreal); cells were regularly tested for mycoplasma (every six months) by Surrey Diagnostics, UK. Expression plasmids containing an exogenous signal peptide of murine origin, the complete extracellular domain of the protein of interest, a recognition sequence for enzymatic biotinylation by the BirA biotin ligase and a 6-His tag were transiently transfected into HEK cells as previously described ^29, 30^. Where expression of the recombinant protein could not be achieved, a rat Cd4d3+4 tag was inserted in the expression construct between the sequence encoding the ectodomain of interest and the biotinylation sequence, which can facilitate expression and secretion of the recombinant protein ^31^. At 6 days after transfection, cell culture supernatants were collected, filtered and supplemented with 20 mM imidazole and 400 mM NaCl before being affinity-purified on HisTrap HP purification columns (GE Healthcare) using an AKTAPure instrument, according to the manufacturer’s instructions. The purified protein fractions were dialysed in phosphate-buffered saline (PBS) and quantified by optical density measurement at 280 nm. To check for purity and integrity of the purified proteins, a 2-µg sample was resolved by SDS-PAGE on NuPAGE 4–12% Bis-Tris polyacrylamide gels (ThermoFisher) for 50 minutes at 200 V. The gels were then stained with SYPRO Orange (ThermoFisher), destained in 7.5% acetic acid and imaged using a Typhoon 9400 phosphoimager (GE Healthcare). The remainder of each purified protein was aliquoted and stored at -20°C until the day of immunisation. These gels are shown in *Underlying data* ^32^.

**Table 1.** Immunisation of BALB/C female mice with 96 recombinant *Schistosoma mansoni* proteins produced in mammalian cells. The protein number, accession number, molecular mass (MM) in kiloDaltons, presence of a rat Cd4d3+4 tag on the antigen, antigen doses and half-maximal serum antibody titres are indicated. Rows shaded in grey correspond to proteins that could not be produced or could not be purified in sufficient amounts for immunisations. Where proteins were produced without and with a Cd4 tag, the molecular mass of the untagged form is indicated first.

Number	UniProt accession number	MM	Cd4 tag	Antigen dose	Half-maximal titre (1:x)
Cell surface					
1	Smp_195190	13	-	100-20-20	8,851
2	Smp_081920	21	-	100-20-20	10,023
3	Smp_166340	24	-	100-20-20	147,432
4	Smp_017730	233	-	100-20-20	331,183
5	Smp_127820	108	-	100-20-20	447,516
6	Smp_194920	70	-	100-20-20	499,904
7	Smp_011680		-		
8	Smp_054070	40	-	100-20-20	1,257,140
9	Smp_073400	45	-	100-20-20	73,944
10	Smp_105220	16	-	100-20-20	188,373/48,073 (repeat)
11	Smp_019350	8/38	-/+	100-20-20	397(-)/38,066 (+)
12	Smp_021220	30	-	100-20-20	34,117
13	Smp_031880	35	-	100-20-20	507,714
14	Smp_009830	48	+	45-20-20	8,717
15	Smp_032520	40	-	100-20-20	233,184
16	Smp_074000	56	+	100-20-20	41,910
17	Smp_102480	14/37	-/+	100-20-20	39(-)/71,705(+)
18	Smp_124000	15/38	-/+	100-20-20	62(-)/15,405(+)
19	Smp_156270	48	+	100-20-20	125,921
20	Smp_174580	35	-	100-20-20	1,017,180
21	Smp_176020	10/34	-/+	100-20-20 (-)/75-20-20 (+)	0(-)/1,284(+)
22	Smp_048380	73	+	100-20-20	374,183
23	Smp_060570	85	+	100-20-20	11,048
24	Smp_075280	45	-	100-20-20	122,414
25	Smp_129840	183	-	100-20-20	350,096
26	Smp_133270				
27	Smp_145420				
28	Smp_149740	97	-	100-20-20	239,927
29	Smp_155810	160	-	100-20-20	69,118
30	Smp_162520	162	+	30-20-20	0
31	Smp_164760	160	-	50-20-20	195,225
32	Smp_166300	70	-	100-20-20	161,156
33	Smp_168240	45	-	100-20-20	332,665
34	Smp_171460	138	-	80-20-20	181,682
35	Smp_176540	123	-	100-20-20	165,471
36	Smp_157070	80	+	100-20-20	82,856
37	Smp_165440	51	-	100-20-20	61,012
38	Smp_136690	75	-	100-20-20	378,652
39	Smp_061970				
40	Smp_153390	71	-	100-20-20	1,379,630
41	Smp_072190	37	-	100-20-20	277,781
42	Smp_064430	24	-	100-20-20	201,941
Secreted adhesion/growth factor/metabolite binding					
43	Smp_194840	24	-	100-20-20	1,048,470
44	Smp_194910	28	-	100-20-20	329,926
45	Smp_063530	26	-	100-20-20	49,886
46	Smp_141680	87	-	90-20-20	535,508
47	Smp_043650	37	+	100-20-20	84,269
48	Smp_170550	140	-	100-20-20	379,757
49	Smp_035040	40	-	100-20-20	62,272
50	Smp_052660				
51	Smp_128590				
52	Smp_135210				
53	Smp_136320	24	-	100-20-20	72,442
54	Smp_144130	118	+	100-20-20	129,754
55	Smp_154760	274	-	100-20-20	356,003
56	Smp_171780	37	-	100-20-20	158,384
57	Smp_178740				
58	Smp_180600	48	-	100-20-20	489,748/347,980 (repeat)
59	Smp_181220	27	-	100-20-20	20,622
60	Smp_211020				
61	Smp_016490	29	-	100-20-20	52,662
62	Smp_130100				
63	Smp_105420	30	-	100-20-20	265,183
64	Smp_105450	34	-	100-20-20	14,672
65	Smp_202610	23	-	100-20-20	109,506
Secreted protease/protease inhibitor					
66	Smp_090100	104	+	20-10-10	2,243
67	Smp_067060	48	-	100-20-20	198,630
68	Smp_103610	49	-	50-20-20	14,029
69	Smp_019030	68	-	80-20-20	117,774
70	Smp_002600	111	+	80-20-20	0
71	Smp_071610	75	-	100-20-20	498,134
72	Smp_089670	386	-	100-20-20	97,493
73	Smp_112090				
74	Smp_119130				
75	Smp_002150	97	+	20-10-10	19,799
76	Smp_141610	49	-	100-20-20	57,720
77	Smp_147730	24/47	-/+	100-20-20	1,008(-)/9470(+)
78	Smp_034420	33	-	100-20-20	68,400
79	Smp_075800	59	-	100-20-20	268,007
80	Smp_210500	52	-	100-20-20	478,317
81	Smp_132480	56/79	-/+	60-20-20(-)/55-20-20(+)	31,288(-)/0(+)
82	Smp_166280	55	-	100-20-20	423,594
83	Smp_187140	50	-	20-10-10	0
84	Smp_006510				
85	Smp_090110				
Secreted enzyme (not protease)					
86	Smp_145920	71	+	80-20-20	0
87	Smp_040790	30	-	100-20-20	303,955
88	Smp_008320				
89	Smp_021730	52	+	100-20-20	18,946
90	Smp_078800	49	+	100-20-20	86,998
91	Smp_007450	25	-	80-20-20	86,888
92	Smp_026930	55	-	100-20-20	311,900
93	Smp_059910				
94	Smp_089240				
95	Smp_134800	243	+	40-20-20	25,828
96	Smp_018760	129	-	100-20-20	59,257
Secreted venom/secreted MEGs/short					
97	Smp_194860	39	+	50-20-20	6,595
98	Smp_138080	30	-	100-20-20	44,679
99	Smp_194830	13/37	-/+	100-20-20	1,964(-)/36,345(+)
100	Smp_001890	32	-	100-20-20	121,269
101	Smp_002070	34	-	100-20-20	202,207
102	Smp_138060	23	-	100-20-20	18,384
103	Smp_180620	18/41	-/+	100-20-20	0(-)/25,592(+)
104	Smp_123540	37/57	-/+	60-20-20 (- and +)	276,631(-)/292,600(+)
105	Smp_123550	42	-	100-20-20	247,541
Secreted - no known function					
106	Smp_181070	43	+	20-20-20	54,950
107	Smp_004710	42	+	100-20-20	43,386
108	Smp_061310				
109	Smp_005060				
110	Smp_141500	56	+	100-20-20	47,639
111	Smp_006060	69	-	100-20-20	338,372
112	Smp_063330	25	-	100-20-20	237,942
113	Smp_096790	38	+	100-20-20	38,574
114	Smp_201730	38	+	100-20-20	74,319
115	Smp_019000	29/53	-/+	100-20-20	0(-)/1,135(+)

-/+: antigens without the Cd4 tag that did not raise high levels of antibodies were produced a second time as Cd4-tagged proteins and immunised in a separate group of animals. For most antigens, mice were first immunised with 100 µg antigen followed by two fortnightly injections of 20 µg protein (100-20-20). Where amounts of purified antigens were not sufficient to follow this dosage, lower amounts of antigens were used as indicated in the table. (-) or (+) indicates results obtained in the absence or presence of a Cd4 tag on the antigen, respectively. In cases of proteins 10 and 58, antibody titres where immunisations were repeated on larger cohorts of animals are indicated (repeat).

To prepare biotinylated antigens for determination of antibody titres, the BirA biotin ligase was co-transfected at the same time as the expression constructs described above into HEK-293-E or -6E cells as previously described ^33^. Supernatants were collected six days post-transfection, dialysed in HEPES-buffered saline, and their level of expression determined by ELISA as previously described ^29^.

### Maintenance of the *S. mansoni* parasite life cycle and preparation of cercariae

All animal experiments were performed in accordance with UK Home Office regulations under project licenses P77E8A062 and PD3DA8D1F, and following approval from the local Animal Welfare and Ethical Review Body. The life cycle of the NMRI (Puerto Rican) strain of *Schistosoma mansoni* was maintained by routine infections of BALB/C female mice and *Biomphalaria glabrata* snails. Infected snails were housed in aerated tanks kept in dark cupboards maintained at 28°C. For collection of cercariae, 20 to 50 infected snails were individually washed in conditioned water, pooled in a small glass beaker, submerged in conditioned water and exposed to bright light ^34^. After 60 minutes, the conditioned water containing live cercariae was collected. To evaluate the concentration of cercariae in solution, 12 5-µL aliquots were sampled, mixed with Lugol (Sigma Aldrich), and cercariae contained in each individual drop were counted under a dissecting microscope. For infection challenges, the cercarial solution was subsequently diluted in conditioned water to a final concentration of 400 cercariae/mL, and immediately used for percutaneous infection.

### Immunisation and percutaneous infection of BALB/C mice

Mice were obtained from the Research Support Facility, Sanger Institute and typically had a mass of 25 g. Mice were housed in GM500 Individually Ventilated Cages or IsoCage N - Biocontainment Systems (Tecniplast) and maintained on individual air handling units kept at between 19 to 23°C and 45–65% humidity; each cage received 60 air volume changes per hour. Mice were given access to food and water ad libitum and maintained on a 12-hour light/dark cycle. Aspen wood chip was used as the base bedding with nestlets and "fun tunnels" for environmental enrichment (Datesand). Mice were housed in groups of no more than 6 adults per cage. Welfare assessments were carried out daily as part of the morning check. This involved moving the cage forward on the rack runners and observing the mice in their micro-environment. Any abnormal signs of behaviour or physical clinical signs of concern were reported. "In hand" weekly health assessments were also performed, and every cage was removed from the rack and each mouse given a full "head to toe" health check. All persons performing welfare checks on animals were trained and assessed as competent by qualified individuals.

For each antigen studied and on the day of immunisation, aliquots of purified protein were thawed, diluted in PBS and mixed 50% v/v with Alhydrogel adjuvant 2% (InvivoGen) for 2 hours at room temperature. For each antigen, a group of five 7-week old female BALB/C mice was immunised intraperitoneally initially with 100 µg protein followed by two additional fortnightly immunisations using 20 µg protein. Where the quantity of purified antigen was insufficient to achieve these levels, lower doses of proteins were administered, as described in Table 1. In each group, a sixth animal was injected with PBS mixed 50% v/v with Alhydrogel 2% and used as a cage control.

After a 4-week rest period, animals were challenged with 200 *S. mansoni* cercariae for 40 minutes by percutaneous infection through the tail. For this purpose, test tubes containing 5.5 mL conditioned water were prefilled and placed onto a bespoke anaesthesia rig (see below) pre-warmed to a surface temperature of 32°C. Mice were anaesthetised in an induction box using 4% isoflurane; 1 L/min oxygen, and an eye ointment was used to prevent corneal damage. After induction of anaesthesia, the animals’ tails were wiped with a tissue soaked with conditioned water to remove any contaminants from the cage bedding that could inhibit cercarial penetration, immersed once more in conditioned water before carefully transferring the animals onto individual holders on the rigs and inserting their tails into the test tubes. Nose cone positions were adjusted for each animal, and anaesthesia was maintained at 2% isoflurane; 1L/min oxygen. Individual splash guards were put in place to reduce the possibility of human exposure to infected water. In each test tube, 500 µL of a stock solution containing 400 cercariae/mL was added. After 40 minutes, animals were removed from the anaesthesia rigs, placed back into their cage and monitored until full recovery from the anaesthesia. At 42 days after the challenge, mice were euthanised by intraperitoneal injection of 200 µL of 200 mg/mL pentobarbital supplemented with 100 U/mL heparin; adult worms were recovered after portal vein section and intracardiac perfusion with phenol-red-free DMEM supplemented with 10 U/mL heparin, and whole livers were collected. Worms collected from individual mice were washed three times in PBS and placed in 6-well plates for counting. Liver tissues were weighed and then digested by incubation in 10 mL of 4% potassium hydroxide for 15 hours at 37°C ^35^. The number of eggs per gram of liver was calculated after counting the number of eggs from 12 10-µL aliquots from each liver homogenate.

### Determination of antibody titres by ELISA

At 8 days after the last immunisation, blood samples (20 to 50 µL) were collected by tail bleed from each animal and left to clot for two hours at room temperature. After centrifugation, sera were collected and stored at -20°C after addition of 2 mM sodium azide. To determine the antibody titre against an antigen of interest, individual sera were serially diluted 1:4 between a 1:2,000 and a 1:8,192,000 dilutions in HBST/2% BSA. Depending on the presence or absence of a Cd4 tag in the antigen studied, these serial dilutions were pre-incubated overnight at room temperature with 1 mg/mL or 100 µg/mL of the rat Cd4d3+4-BLH protein, respectively, to block the fraction of antibodies that were raised against the tag in immunised animals. Serially diluted sera were then transferred to streptavidin-coated ELISA plates on which the biotinylated antigen of interest had been immobilised. To ensure that the antibody fraction directed against the protein tag had been blocked, binding of the lowest dilution of antisera was also tested against the control rat Cd4d3+4-BLH protein immobilised on an ELISA plate to confirm absence of anti-tag reactivity. Detection was performed using an alkaline-phosphatase anti-mouse immunoglobulin secondary antibody followed by incubation with an alkaline phosphatase substrate, as previously described ^29^.

### Statistical analysis

Statistical analysis was performed using GraphPad Prism (version 5) software. Data from serum analysis were analysed using a non-linear regression model to determine half-maximal antibody titres. R ^2^ scores between the number of eggs per gram of liver and the total number of adults or the total number of mature females were calculated by linear regression analysis. Comparison of average numbers of eggs per gram of liver between groups of immunised animals in a same cohort was performed using one-way ANOVA analysis followed by Bonferroni correction. Where immunisations were repeated on larger cohorts of animals, an unpaired t-test was used to compare average numbers of eggs in immunised and control animals; P-values ≤ 0.05 were considered to be significant.

## Results

### Optimising the conditions for percutaneous infections by *S. mansoni* in mice

**Figure 1.**
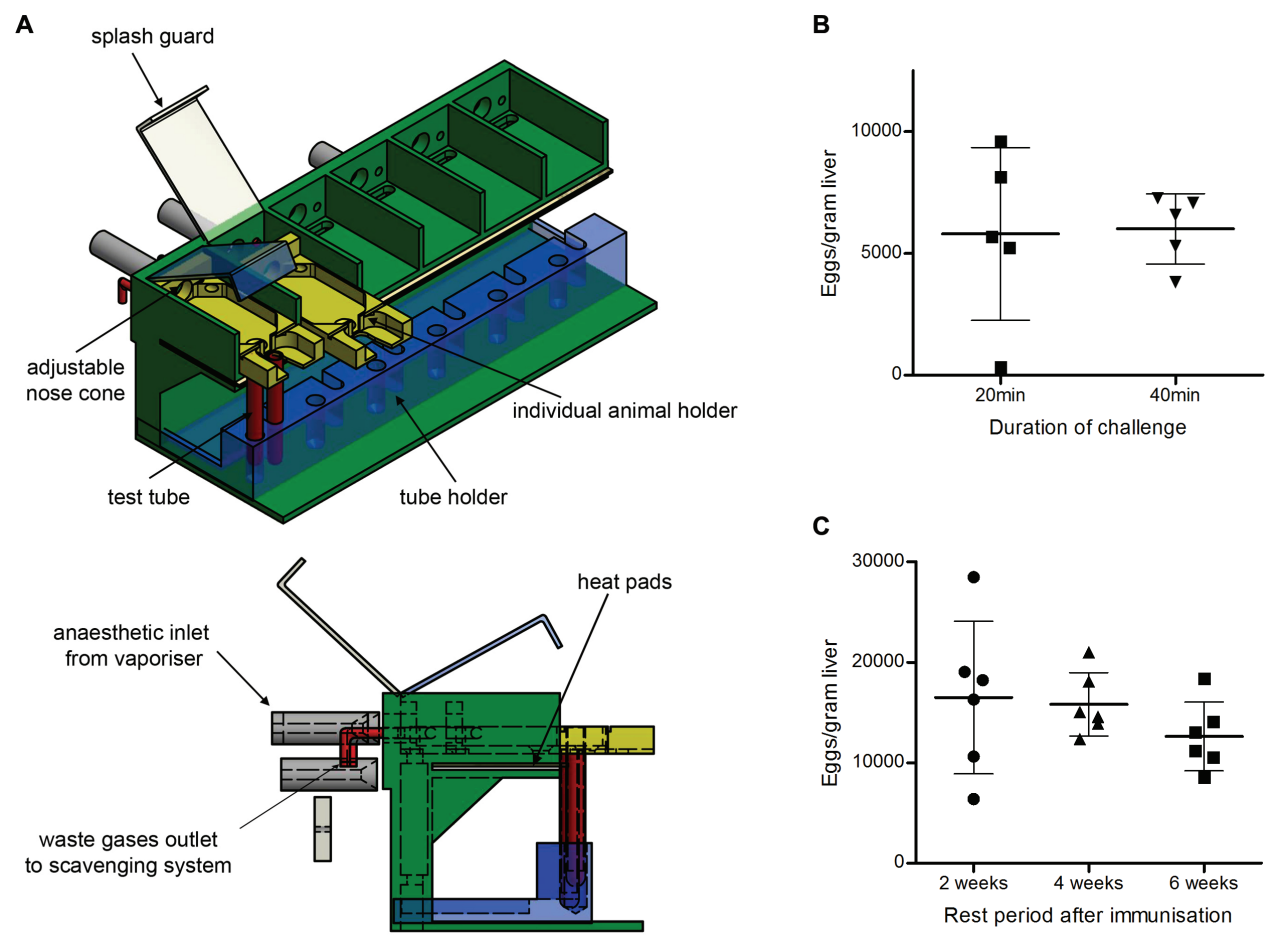
Anaesthesia rig for percutaneous infection of mice with *Schistosoma* parasites and challenge optimisation. ( **A**) Each anaesthesia rig is divided into six individual compartments, each with their own adjustable nose-cone, splash guard and animal holder (yellow) for the safe removal of animals after the challenge. Test tubes (red), containing cercariae-infected water, are placed onto a removable tube holder (blue). Surface temperature of the instrument is maintained at 32°C using heat pads located underneath each compartment and controlled by a heat controller and temperature sensors (not shown on the figure). ( **B**) 12-week-old BALB/C female mice were challenged with 200 *S. mansoni* cercariae from the same parasite preparation for either 20 minutes ( *n* = 5 animals) or 40 minutes ( *n* = 5). The numbers of eggs per gram of liver were counted 42 days later from whole liver preparations from each animal. Inter-individual variability was greater between animals that had been challenged for 20 minutes compared to those challenged for 40 minutes. ( **C**) Three groups of 6-week-old BALB/C female mice were immunised three times fortnightly with 100 µg of a control protein in 1% Alhydrogel. The first group ( *n* = 6 animals) went through a 2-week rest period between their final immunisation and the infection challenge with 200 *S. mansoni* cercariae, while the second group ( *n* = 6) had a 4-week rest period and the third group ( *n* = 6) had a 6-week rest period. The numbers of eggs per gram of liver were counted 42 days later from whole liver preparations from each animal. No statistically significant difference in the average number of eggs could be identified between the three groups of animals. Note that the group of animals with a four-week rest period was infected with a different cercarial preparation from the other two groups.

To compare the protective efficacy of a large number of antigens in a systematic manner, we first decided to optimise the experimental conditions of *S. mansoni* infection in mice. These are usually done either by intra-peritoneal injection of cercariae or by exposure of the skin to the parasite (percutaneous infection) ^34^. Because a large fraction of the antigens to be tested correspond to parasite proteins expressed in the early dermal stages of infection, we opted to use the percutaneous method, which is also a natural route of infection. This protocol, however, requires a relatively lengthy exposure of either restrained or anaesthetised animals to infested water. To minimise unnecessary stress to the animal and biological safety risks to the experimentalist, we designed a bespoke anaesthesia apparatus that allowed for prolonged exposure to the parasite by tail immersion into cercariae-containing water while animals were under the influence of a gaseous anaesthetic ( Figure 1A). The instruments were fitted with an inlet connected to a vaporiser for delivery of anaesthetics, and an outlet linked to a scavenging system for disposal of waste gases. Each device could accommodate up to six animals and was divided into six individual compartments, each with its own holder, splash guard, adjustable nose cone and test tube holder for immersion of the animals’ tails into cercariae-containing water. To ensure animals did not suffer from hypothermia during anaesthesia, the instruments were fitted with heat-pads, a heat controller and temperature sensors and the surface temperature maintained to 32°C throughout the experiment. All of the infection challenges described in this study were performed using these devices.

As mouse infection by *Schistosoma* parasites can be affected by the presence of testosterone ^36^, we decided to use female mice of the BALB/C strain, which have a robust Th2-type immune response ^37^. To determine the optimal duration of the infection challenge, two groups of animals were infected with the same preparation of cercariae for either 20 or 40 minutes and their infection levels measured six weeks later by counting the number of eggs per gram of liver. Although all challenged animals were infected, the inter-individual variability was smaller in the group exposed for 40 minutes ( Figure 1B). Increasing the infective dose to 300 or 400 cercariae for 40 minutes led to an increase in premature mortality around three weeks post-infection (two out of six mice had to be euthanised prematurely in the group challenged with 300 cercariae; and five out of five in the group challenged with 400 cercariae). Based on these observations, infection challenges were standardly performed by immersing the tail of anaesthetised mice in 6 mL of conditioned water containing 200 *S. mansoni* cercariae for 40 minutes.

To elicit high levels of antibodies against specific antigens, we opted to use Alhydrogel, a relatively mild alum-based human-compatible adjuvant, which promotes a Th2-type host response ^38^. In some vaccine experiments, administration of adjuvant alone can also confer some protective effect, possibly through the general stimulation of the host immune system. To determine whether Alhydrogel could cause a so-called “adjuvant effect”, we immunised three groups of animals three times fortnightly with a control protein in 1% Alhydrogel before challenging them two, four or six weeks post-immunisation. No statistically significant difference in the average infection levels could be observed between the three groups of animals, although the inter-individual variability was greater in the group challenged two weeks after the final boost ( Figure 1C). Based on these observations and to reduce the lead time of each experiment, we decided to adopt a 4-week rest period between the final immunisation and the infection challenge. Raw data for optimisation of immunisation and rest periods are available as *Underlying data* ^32^.

### Cell-surface and secreted *S. mansoni* antigens generate a strong host immune response

**Figure 2.**
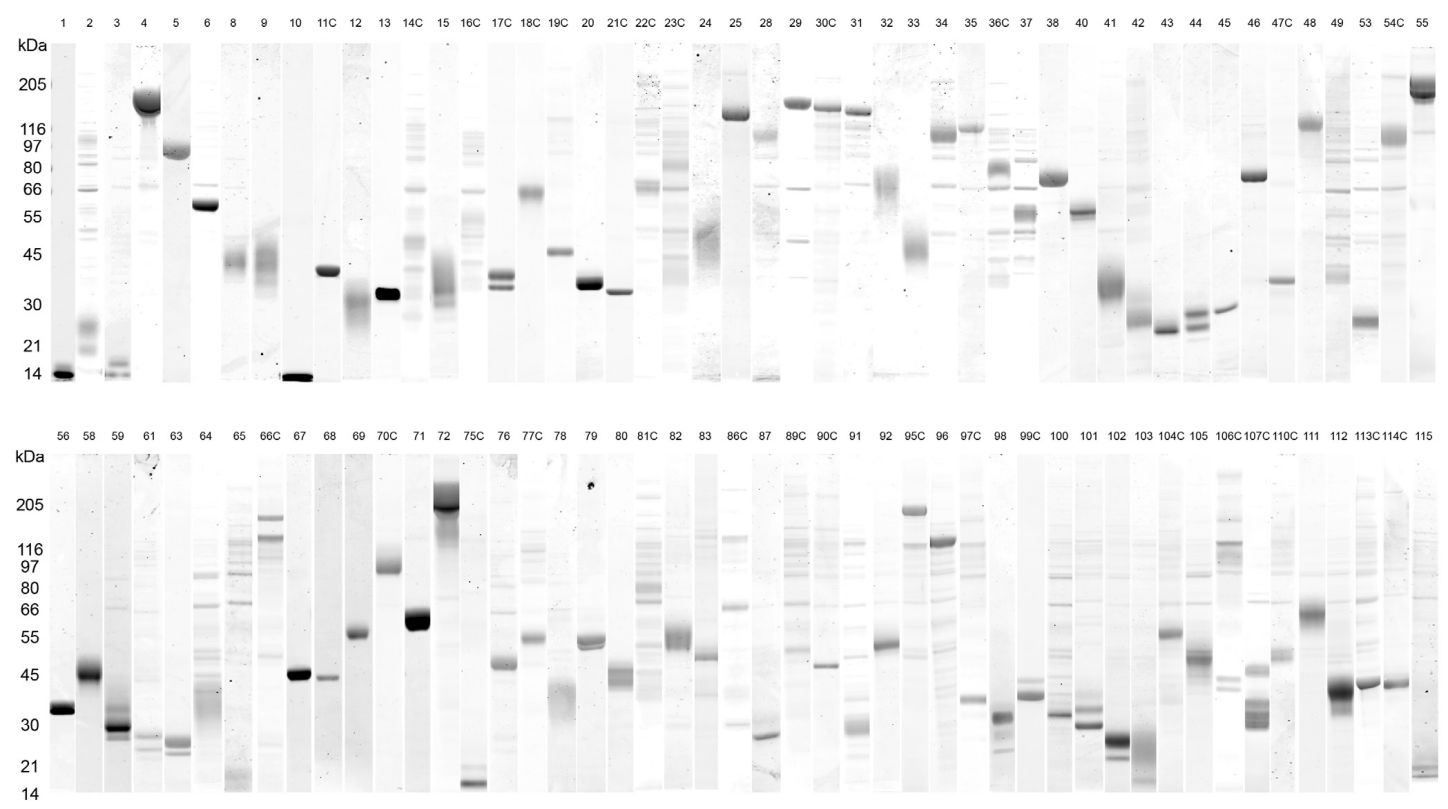
The 96 recombinant cell-surface or secreted *Schistosoma mansoni* antigens produced in mammalian HEK-293E cells. Proteins were purified by Ni ^2+^-affinity purification using their carboxy-terminal 6-His tag and a small aliquot (2 µg) resolved by SDS-PAGE. Numbering of the proteins corresponds to Table 1. The suffix C indicates antigens that were produced with an additional rat Cd4d3+4 tag at their C-terminal end. Proteins were typically heterogeneous in mass, reflecting the presence of different glycoforms in the preparation. In the case of low-expressing proteins, contaminants originating from the cell culture supernatant occasionally co-purified alongside the protein of interest and appear as fainter background bands on the gels. Molecular masses (kDa) are indicated on the left-hand side of each composite picture. Adjustments to brightness and contrast for individual proteins were performed using the Adobe Photoshop CS5 software to improve clarity.

**Figure 3.**
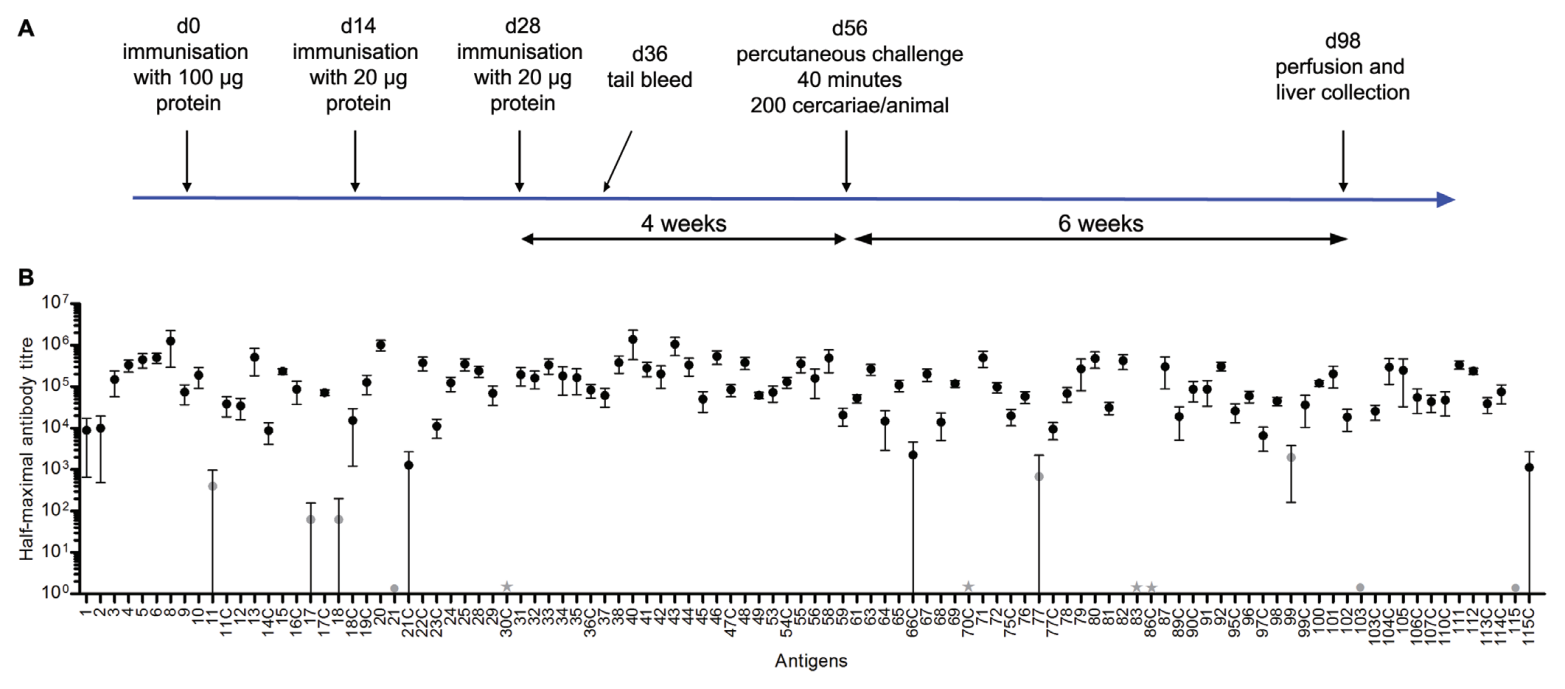
Recombinant cell-surface and secreted *Schistosoma mansoni* antigens raised robust immune responses in mice. ( **A**) Experimental timeline detailing the immunisation regime, blood sampling, percutaneous infection and sample collection followed for each group of immunised animals. ( **B**) The average half-maximal antibody titres of mice immunised with individual antigens are shown. The suffix C indicates the presence of a C-terminal rat Cd4d3+4 tag in the antigen tested. Protein numbering corresponds to that presented in Table 1. Antigens that were initially tested without a Cd4 tag and induced half-maximal titres <1:10,000 are shown as grey circles. Four antigens (proteins 30C, 70C, 83 and 86C) did not induce any humoral response and are indicated by grey stars. Datapoints represent mean ± SD; *n* = 5 or more animals per group, with the exception of proteins 13, 18C, 28 and 42 ( *n* = 4), and protein 17C ( *n* = 3) due to premature mortality.

We have recently described the selection of a panel of *S. mansoni* cell-surface and secreted proteins identified from proteomics and transcriptional studies, of which 103 could be produced recombinantly in human embryonic kidney (HEK)-293 cells as soluble ectodomains containing a C-terminal rat Cd4d3+4 tag followed by a sequence for enzymatic monobiotinylation and a 6-His tag for purification ^28^. The majority of these proteins were immunoreactive when tested against sera from *S. mansoni-*infected patients living in areas of high endemicity, and contained heat-labile epitopes demonstrating the presence of conformational epitopes. We attempted to produce all 103 antigens as soluble 6-His-tagged proteins (without the C-terminal rat Cd4 tag) and managed to obtain 74 antigens in quantities sufficient to perform immunisations in mice. For another 22 antigens, sufficient expression levels could only be achieved when these proteins were produced as C-terminally Cd4-tagged and 6-His-tagged antigens. Finally, seven proteins (Proteins 7, 51, 52, 57, 62, 88 and 108) were not expressed at sufficient levels to perform immunisations in either form ( Table 1). All 96 proteins were purified by Ni ^2+^ affinity-purification and the integrity of all purified proteins was determined on protein gels ( Figure 2); these included very large proteins such as protein 55 (274 kDa) or protein 72 (386 kDa). Given the large number of antigens to be tested and because we were attempting to identify protective candidates conferring a large effect size, we performed the immunisations and challenges in cohorts, each comprising of three groups of one control and five experimental animals, where each group was vaccinated with a different protein. For all but 18 antigens, sufficient amounts of protein were produced to immunise the mice with one initial injection of 100 µg protein per animal followed by two subsequent 20-µg boosts. Where protein expression was not sufficient to achieve this regime, lower doses of antigens were administered as described in Table 1. At 8 days after their final immunisation, blood samples were taken from each animal and the antibody titres determined by ELISA ( Figure 3A). We arbitrarily decided that average half-maximal antibody titres >1:10,000 constituted “good” immune responses from the groups of animals tested, while titres <1:2,000 were considered as “insufficient”. Eight proteins produced without a Cd4d3+4 tag raised insufficient immune responses, resulting in average half-maximal titres lower than our arbitrary cut-off of 1:2,000 (Proteins 11, 17, 18, 21, 77, 99, 103 and 115). In these instances, the same proteins were produced again in their Cd4-tagged form, and immunisations were repeated on different groups of animals. This approach generally improved the antigenicity of the ectodomains of interest: in five out of eight cases, reactivity to the recombinant parasite ectodomains resulted in average half-maximal titres greater than 1:10,000. Overall, most recombinant parasite proteins proved to be antigenic with all but 11 antigens giving half-maximal titres greater than 1:10,000 (89%, Table 1, Figure 3B). Of the 11 antigens with lower reactivity, four produced no immune response (Proteins 30C, 70C, 83 and 86C). Antibody titres are available as *Underlying data* ^32^.

### A systematic challenge of immunised animals with *S. mansoni* does not identify strongly protective antigens

To determine whether any of the antigens tested had a protective effect against infection by *Schistosoma mansoni*, each group of immunised animals was challenged four weeks after their final immunisation with 200 cercariae for 40 minutes. Because preparations of cercariae differed in their infective efficiency, each batch of parasite was used to simultaneously challenge cohorts of three groups of animals, each immunised with a different protein. In total, 36 cohorts were challenged to test the protective efficacy of 96 recombinant *S. mansoni* proteins.

**Figure 4.**
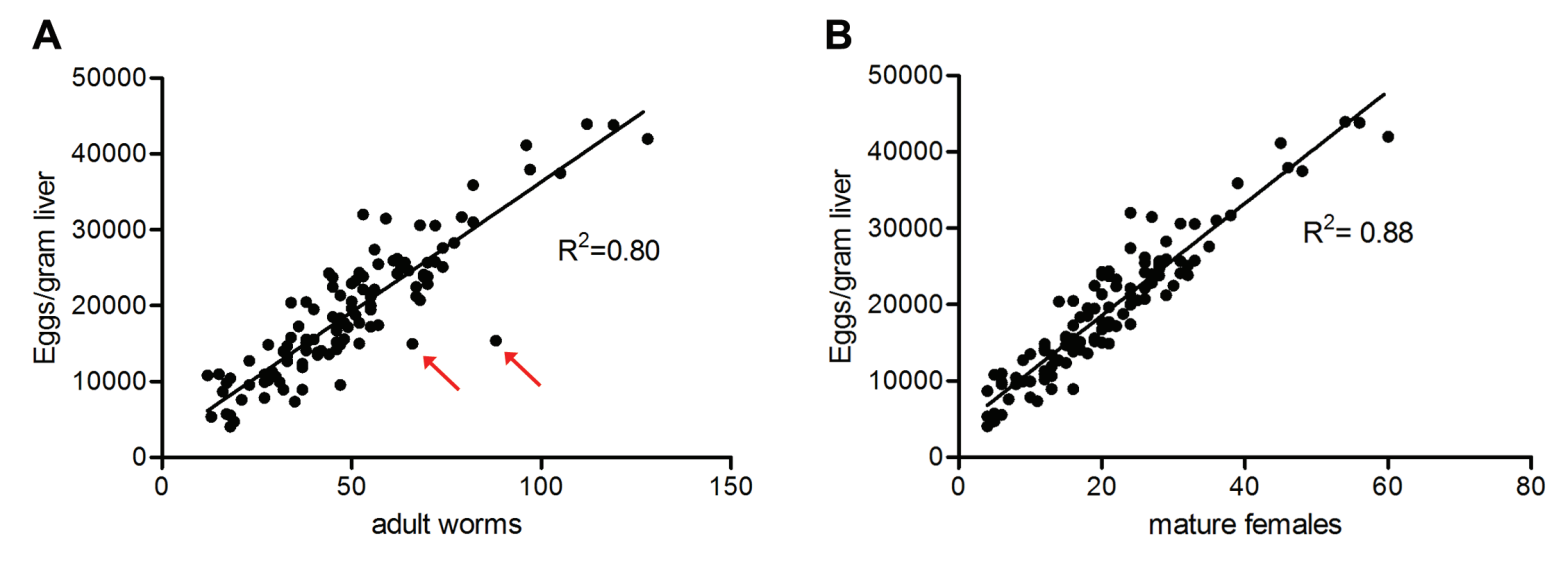
The number of eggs per gram of liver correlates with the number of adult worms. ( **A**) For each group of immunised animals, the average number of eggs per gram of liver was plotted against the average number of *S. mansoni* worms per animal recovered after perfusion. A high correlation (R ^2^ = 0.80) was observed between the two measurements. Red arrows indicate groups of animals immunised with proteins 98 and 100, in which a marked deficit in the number of eggs compared to the total number of adult worms was observed. This was attributed to a sex ratio imbalance in the cercarial preparation used for the infection challenge, resulting in an excess of (mostly sexually immature) female worms. Across all groups of animals tested, variability in parasite recovery ranged from 6 to 64%, reflecting the heterogeneity in the cercarial preparations used to infect the different cohorts of animals. ( **B**) For each group of immunised animals, the average number of eggs per gram of liver was plotted against the average number of mature female worms per animal recovered after perfusion. A high correlation was observed between both measurements (R ^2^ = 0.88).

At 42 days after infection, the animals were euthanised, perfused, and their livers were collected. Two parameters were measured to determine the levels of infection: the total number of worms recovered after perfusion, and the number of eggs per gram of liver. In one cohort, the infection challenge failed as even the PBS-immunised control animals from each cage in this cohort showed no sign of infection, and mice immunised with proteins 14C and 23C were therefore excluded from the study. Overall, we observed a good correlation between the number of adult worms and the number of eggs per gram of liver (R ^2^= 0.80; Figure 4A) across all groups of immunised animals tested, and this correlation improved further if only morphologically mature adult females were taken into consideration (R ^2^= 0.88; Figure 4B). However, we observed significant variability in infection efficacy between the batches of cercariae used: the percentage of parasite recovery ranged from 6 to 64% ( Figure 4A) and on average, the percentage recovery of adult worms across all groups studied was 25%. In the few cases where a marked deficit in the number of eggs was observed when compared to the total number of adult worms (proteins 98 and 100, Figure 4A), we noticed a similar phenomenon in the control animals from this cohort. This was attributed to a strong sex ratio imbalance in the cercarial preparation used to challenge the cohort, resulting in a vast excess of immature females.

**Figure 5.**
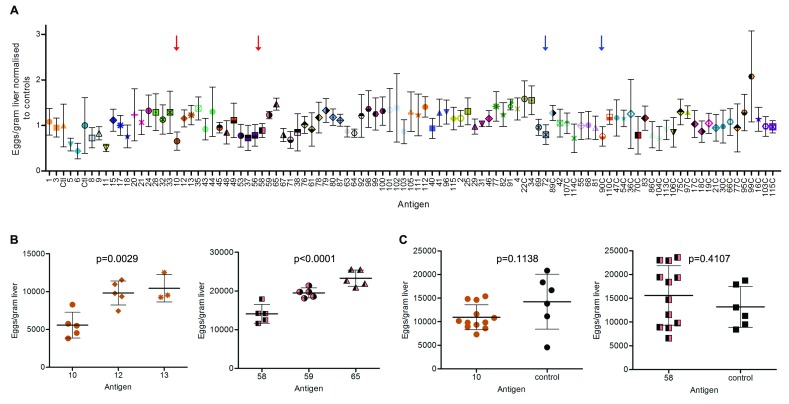
Systematic challenge of animals immunised with *Schistosoma mansoni* antigens does not identify strongly protective candidates. ( **A**) An overview of the 36 cohorts of immunised animals challenged with *S. mansoni* cercariae. Each cohort is represented by a different colour and is composed of two or three groups of animals. For each group of immunised animals, the average number of eggs per gram of liver has been normalised to the average number of eggs per gram of liver of the PBS-immunised controls from the same cohort. Red or blue arrows indicate groups of animals that showed statistically significant difference from two or one group of the same cohort, respectively. Datapoints are mean ± SD; *n* = 5 or more animals per group, with the exception of proteins 17, 18C, 28, 40, 55, 56, 64, 66C, 69, 81, 87, 99C and 102 ( *n* = 4), and proteins 13, 17C and 42 ( *n* = 3) due to premature mortality. ( **B**) Details of individual datapoints in cohorts corresponding to antigens 10 and 58, for which a statistically significant difference in the number of eggs per gram of liver was observed by ANOVA analysis compared to the other groups from the same cohort (p=0.0029 and p<0.0001, respectively). Bars are mean ± SD; *n* = 5 animals per group, with the exception of protein 13 due to premature mortality. ( **C**) Repeat experiments with antigens 10 and 58 on larger cohorts of animals shows no statistically significant difference between animals immunised with the antigen of interest ( *n* = 12) compared to animals who received PBS ( *n* = 6). Individual datapoints are shown. Bars are mean ± SD.

Given the good overall correlation between the average number of worms recovered after perfusion and the average number of eggs per gram of liver observed in the groups studied, we based our next analysis on the number of eggs per gram of liver recovered after whole liver digestion. This also presented with the added advantage of measuring the level of infection as well as any effect an antigen could have on female worm fecundity. A summary of all cohorts studied is presented in Figure 5A. To identify statistically significant differences between groups of animals within the same cohort - which were all infected with the same batch of cercariae - the numbers of eggs per gram of liver from each of the three groups of immunised animals were compared to each other by ANOVA analysis and Bonferroni correction. Using this approach, animals immunised with proteins 10 (Smp_105220) and 58 (Smp_180600) showed a statistically significant reduction by ANOVA analysis (p=0.0029 and p<0.0001, respectively) when compared to the other two groups from their respective cohorts ( Figure 5B), while those immunised with proteins 72 (Smp_089670) and 90C (Smp_078800) showed a statistically significant reduction (p<0.05) compared to one group from their cohort.

To confirm these observations, we repeated the immunisations for proteins 10 and 58 on a larger number of animals. Three cages each containing four animals immunised with the protein of interest ( *n* = 12) and two PBS-immunised controls ( *n* = 6) were compared. In both repeats, the average half-maximal antibody titres were lower than in the primary screen at 1:48,073 for protein 10 and 1:347,980 for protein 58. We did not see any statistical difference between control animals and those immunised with protein 58 in this larger cohort. With the exception of one control animal, which only had a few mature females and therefore had a much lower egg burden, we once again observed a trend towards a reduced number of eggs per gram of liver in animals immunised with protein 10 compared to controls, although this observation did not reach statistical significance ( Figure 5C). All values concerning egg counts are available as *Underlying data* ^32^.

## Discussion

Schistosomiasis is a debilitating parasitic disease that plagues the lives of millions of individuals across the globe. With 10% of the world population living in areas where schistosomiasis is endemic and more than 70 million people needing treatment every year, the development of new preventative methods remains a priority. Mass administration of praziquantel to populations at risk of contracting the disease is, however, the most common method of prevention and treatment in human populations, and very few vaccine candidates have progressed to clinical trials so far. In this study, we have systematically compared the protective efficacy of 96 cell-surface and secreted antigens from *S. mansoni* produced in a mammalian expression system. This is, to our knowledge, the largest set of *Schistosoma* proteins tested in parallel in protection experiments to date.

By using a mammalian expression system, we have sought to improve the tertiary conformation of the recombinant antigens tested, in particular the formation of structurally critical disulphide bonds. Indeed, immunoreactivity experiments on these proteins against patients’ sera from a highly endemic area have shown that the vast majority of them contain heat-labile conformational epitopes ^28^, which are indicative of their correct folding.

Throughout the study, we have adopted very systematic protocols for antigen production, immunisation and infection challenges to limit experimental variability. For example, recombinant proteins were systematically produced in HEK-293 cells and the quality of the purified antigens assessed by SDS-PAGE; strict timings were observed between immunisations (every two weeks) and between the last immunisation and the infection challenge (four weeks); all cercarial preparations were carefully counted and used within one hour of cercarial shedding for infection; the tails of all animals were carefully wiped and immersed in conditioned water before the infection to avoid any inhibitory effect of residual oils present in animal bedding on parasite penetration. Despite these precautions, we still observed significant variability in the efficiency of infection and, in some cases, sex ratio imbalance in the cercarial preparation, which resulted in an excessive number of adult worms compared to the number of eggs produced. One way to better control this imbalance would be to use snails singly infected with male or female miracidia and mix the cercariae produced by these snails in equal quantities.

Of the 96 recombinant candidates that were tested, all but 11 were antigenic enough to generate half-maximal titres > 1:10,000 (our arbitrary criterion for a good humoral response), and only four did not raise any immune response. The lower immunogenicity of some proteins could be attributed to their relatively small sizes (six of them had a molecular mass <50 kDa) or to the lower antigen doses administered during immunisations because of poor expression. Despite overall high reactivities, we could not observe any reproducible protective effect from antibodies raised against the antigens tested. These include proteins that had previously been proposed as vaccine candidates such as Sm29 ^39^ or cathepsin B ^40^. Other promising vaccine candidates such as the intracellular protein Sm-p80 ^41^ or the multimembrane spanning SmTSP2 ^26^ were not included in our screen since their subcellular localisation or structure were not compatible with secretion of the recombinant proteins by HEK-293 cells in the culture supernatant. One possible explanation for the lack of protective efficacy observed in our study could be a biased immune response caused by the adjuvant that we used for immunisations. When designing this systematic study, we selected an adjuvant that could induce a good humoral response in mice but could also be transferable to human vaccine studies. Alhydrogel is a mild aluminium-hydroxide-based adjuvant commonly used in human vaccine formulations, which induces a Th2-type immune response mostly skewed towards the production of IgG1 antibodies ^42, 43^. While this antibody isotype results in high-affinity neutralising antibodies in mouse, it is far less efficient in recruiting complement and binding to activating Fcγ receptors ^44^. The use of alternative adjuvants associated with a more balanced Th1/Th2 immune response such as Quil A might therefore be considered. One alternative explanation could be functional redundancy between some of the proteins tested. Our protein library contains multiple members of large protein families (Ly6, MEG, saposins, cathepsins, VAL, etc) and although these members are immunologically distinct, they could be functionally redundant. Immunisation with mixtures of antigens from the same family of proteins could therefore also be considered.

Of all the candidates that we tested, proteins 10 and 58 showed a statistically significant decrease in the number of eggs per gram of liver in the primary screen when compared to the other two immunised groups from their respective cohort. However, only protein 10 showed a similar trend in the repeat experiment on a larger number of animals, although this did not reach statistical significance. This protein belongs to the Ly6 family of proteins and is also known as SmLy6B/SmCd59b. In previous studies, SmLy6B has been shown to be widely expressed in both male and female worms ^45^ and mice immunised with a DNA vaccine encoding the corresponding gene showed a reduced worm burden close to statistical significance ^15^. Interestingly, the proportion of mature adult females recovered after perfusion in our experiment was similar between immunised and control groups (46.4% adult females in immunised animals versus 45.3% mature females in controls), while the average total number of worms recovered was slightly smaller in the group vaccinated with protein 10 (27 worms versus 33 worms in the PBS controls). Individual cohort data for antigens 10 and 58, in addition to repeat analysis on larger groups, are available as *Underlying data* ^32^.

In conclusion, we have developed a technical infrastructure to refine the conditions of percutaneous infection in mice and to systematically screen a large number of recombinant cell-surface and secreted antigens from *S. mansoni* as vaccine candidates. Although the vast majority of the 96 proteins tested raised a robust immune response in mice, none of them were associated with a strong protective effect against infection by the parasite and the identification of new vaccine candidates against schistosomiasis remains much needed. We hope nonetheless that these antigens will constitute a useful resource for the scientific community and that their role as vaccine candidates will be further explored in the context of different adjuvants, as components of protein mixtures or in other animal models.

## Data availability

Figshare: Data files describing the systemic screening of 96 Schistosoma mansoni cell surface and secreted antigens produced in a mammalian expression system. https://doi.org/10.6084/m9.figshare.9862139.v1 ^32^.

This project contains the following underlying data:

- 1. Optimisation of the duration of challenge for percutaneous infection.pzf
- 2. Optimisation of the rest period after immunisation.pzf
- 3. S. mansoni proteins gels.pptx
- 4. Half maximal antibody titres.pzf
- 5. Correlation Eggs vs Total Adults or Mature Females.pzf
- 6. Eggs per gram of liver counted across cohorts.xlsx
- 7. Cohort analysis - Individual data for antigens 10 and 58.pzf
- 8. Repeat analysis on larger groups - Proteins 10 and 58.pzf
- RDS-SPRN-00358.xlsx (containing all data from datasets 1, 2, 4, 5, 7 and 8, above, in an open format).

Data are available under the terms of the Creative Commons Zero "No rights reserved" data waiver (CC0 1.0 Public domain dedication).
