# Supplementary material for: Systematic screening of 96 Schistosoma mansoni cell-surface and secreted antigens does not identify any strongly protective vaccine candidates in a mouse model of infection
Source: Wellcome Open Res. 2019 Oct 16;4:159. [Version 1] doi: 10.12688/wellcomeopenres.15487.1 (PMC6833992; doi:10.12688/wellcomeopenres.15487.1)
Supplement: Peer review history

# Peer review: Reviewer response for version 1

**DOI:** 10.21956/wellcomeopenres.16943.r36798

**Published:** 2019-11-13

**Authors:** John P. Dalton

**Affiliations:** 1 School of Biological Sciences, Queen's University Belfast, Belfast, UK

The paper by Crosnier et al. represents a tremendous amount of careful and valuable work – the testing of a large panel of vaccines is clearly not an easy task, especially considering the vagaries of the trial system of vaccination followed by challenge infection with live cercariae. The large variation between the infectivity of the cercariae batches used in this paper shows how difficult it is to standardise schistosome vaccine trials. However, to their credit, the authors went to great lengths in animal husbandry, animal housing (e.g. controls in each cage), methodology in infections (including repeat experiments) and analysis of data to ensure that the results they obtained were reliable.

 The results of this paper reveal that vaccination with 96 different S. mansoni parasite molecules, selected based on the expression on the surface of early stage schistosomules or secreted by these (i.e. a large complement of the parasite-host interaction), does not induce protection in mice against a challenge of 200 cercariae. 

 This result leaves myself, and I am sure the authors and members of the schistosome community, a little dazed. Surely, within this wide range of important molecules there should be a least one protective antigen? So how do we explain this result?

- The molecules and their recombinant production: As mentioned above, the 96 molecules were rationally selected because of their putative role in the parasite-host interactome – the most likely molecules that vaccine-induced antibodies could access.  

All molecules were produced in HEK-293 cells to ensure correct folding of the proteins. The production of the same molecules (without a His tag) is described in a previous paper by Crosnier (reference 28 ^1^) who demonstrated by ELISA that the epitopes on the majority of molecules were heat labile – i.e. the purified recombinant bound to antibodies in serum from a pool of infected humans but heat-treated recombinants did not. This does not necessarily prove that the expression system delivers properly folded proteins as heat-treatment can cause precipitation or aggregation of proteins that may affect their binding to ELISA plates. Since the panel of molecules contained a number of proteases and protease inhibitors these could have been tested for functional activity to help validate the expression system.

 The expression system can also add mammalian-like glycans to recombinant proteins. Figure 2 displays an impressive array of purified proteins. Some are well-defined bands while others are smeary and may be glycosylated – it would be useful to know the proportion of proteins that carry mammalian-like glycans.

- The vaccine protocol: The protocol used is standard in the field – 2 to 3 immunisations followed by challenge with cercariae. The challenge infection was given 4 weeks after the final vaccination which goes some way in avoiding spurious protective responses that may operate at the level of the lungs in challenge infections given within a week or two after vaccination (as suggested by Wilson et al 2012, reference 14). 

Alhydrogel was chosen as the adjuvant (mixed 50:50 with antigen solution). It was selected because it can be used in humans. Published data suggests it induced Th2-like responses. Given the failure of all molecules to induce protection, it is possible that this adjuvant is not suitable for eliciting the necessary protective responses against schistosomes in mice and therefore, as the authors point out a different adjuvant(s) would be important to test. A small sub-panel of antigens from each group (surface, secreted) could be selected for adjuvant studies.

 The antigen doses administered to the mice seems very high to me. The first vaccine of 100 ug was followed by two boosts of 20 ug. Could this high quantity of vaccine have induced some immune-tolerance, notwithstanding the high antibody response obtained? Generally, two doses of 20 ug would be sufficient to induce memory responses that would re-boosted following challenge. Perhaps dose-ranging some selected antigens would be useful?

- Antibody responses. The vaccinated mice returned with a ‘robust’ immune response to vaccination i.e. they produced high titre total antibodies (half maximal titre >10,000). It would be informative to know the isotype of antibodies and have some indication of cellular responses to determine if these were similar across all vaccinated groups and, therefore, consisted of a profile of ‘non-protective’ responses (that may correlated with Alhydrogel adjuvant and/or high-dose vaccination, see above).  

Whole-mount immunolocalisation studies could determine if immune responses against surface antigens did induce surface-binding antibodies.

- An interesting finding in this study is that after seven weeks of infection with 200 cercariae (with high variable takes), adult worms numbers correlated with eggs per gram of liver tissue (better correlations were found with female worms versus total worms). This could seem obvious – the more females the more eggs! – but in various vaccines studies reported it is not always the case. 

 It would have been relevant to assess if this correlation held true for intestinal-tissue eggs which are destined to pass on infection to snails, especially since specific immune responses are suggested to aid egg transport through the intestinal wall.

 Nevertheless, does this correlation suggest that the immune responses generated to the vaccines in this study do not neutralise their target and have absolutely no adverse effect on female worms – antigens, such as cathepsin proteases, are involved in worm feeding and it has been suggested that inhibiting this activity would indirectly affect egg production.

- The mouse model. Does this study suggest mice are simply not a good model for testing recombinant schistosome vaccines? Female Balb/c were used in the studies. It may be worthwhile trying selected antigens in the C57/Bl mice given their propensity to elicit a more Th1-like response.

- According to Crosnier (reference 28 ^1^) most of the antigens are not immunogenic in infected mice at 6 weeks post infection. Although saposin-domain and cathepsin B are immunogenic at this time-point these still did not induce protective immune responses. Most antigens were also reactive with antibodies in pooled serum from infected humans, but we do not know if these antibodies have any protective ability. It seems, immunogenicity/non-immunogenicity in normal infections cannot be used to select vaccines 

The irradiated-cercariae vaccine model is mentioned in the introduction of the paper because it consistently induces protection in mice. Do antibodies from these mice preferentially bind any of the 96 molecules? Could this be a criteria for selecting antigen to move forward into adjuvant, dose, mouse strain trials?

  The above points are purely nit-picking to find an ‘generic’ answer to the lack of protective responses found amongst 96 vaccines. I believe this is a milestone paper in schistosome vaccine research, and rather than disappoint the schistosome community it should encourage us to come together to discuss ways of moving forward.

I confirm that I have read this submission and believe that I have an appropriate level of expertise to confirm that it is of an acceptable scientific standard.

## References

[1] : A library of cell-surface and secreted proteins fromSchistosoma mansoni identifies early serological markers of infection. *bioRxiv*.2019; 10.1101/593616 10.1101/593616

# Peer review: Reviewer response for version 1

**DOI:** 10.21956/wellcomeopenres.16943.r36799

**Published:** 2019-11-04

**Authors:** Henry J. McSorley

**Affiliations:** 1 Centre for Inflammation Research, University of Edinburgh, Edinburgh, UK

In this study, the authors express 96 schistosome proteins in a mammalian cell system, and test these in a vaccination study against schistosome infection. No significant reduction in schistosome egg counts was seen with any antigen. However, the technical achievement of successfully expressing and purifying this number of antigens in mammalian cells should not be underestimated, and the setup for reproducibly infecting large numbers of mice with schistosomes for vaccine trials could be used by many other researchers in the future. 

 There are some issues with the experimental setup - it seems a very large set of animal experiments to start, before knowing that any vaccination regime would show an effect. As mentioned in the discussion, several proteins were included in the screen which have already been shown to reduce egg burden after vaccination, eg Sm29 and cathepsin B, however these did not show reduced egg burden using the authors' vaccination regimen. Before commencing a trial of a large number of new candidates, these known protective candidates should have been validated. As pointed out in the discussion, these successful vaccine studies used different adjuvants which induced a more mixed TH1/TH2 response, which could well be more effective than the purely TH2 response induced here by Alhydrogel. Other human-approved adjuvants are available, including Quil A (as mentioned) or Montanide (used in the Cathepsin B study), these could have been tested as part of the optimisation steps.

 To be clear about the potential problems with the experimental setup, I suggest the title should be changed to "Systematic screening of 96 *Schistosoma mansoni* cell-surface and secreted antigens does not identify any strongly protective vaccine candidates in a mouse model of infection, using an Alhydrogel adjuvant". The adjuvant, and potential concerns with it, should also be mentioned in the abstract.

 In Table 1, where antigens are known by another name (e.g. Sm29, cathepsin B) these should be indicated on the table. This would be a useful resource for easily being able to see if a antigen expresses in HEK293 cells, and whether it is immunogenic.

 In Figure 2, it would be useful for the reader to indicate the expected molecular weight of expressed proteins, as in many cases it is unclear which band is the protein of interest, e.g 14C, 16C, 23C.

 In Figure 5A, it would increase the readability to have the groups in numerical order of antigen. This would allow the reader to identify a particular antigen of interest to them more easily. The groups could remain colour-coded by cohort so that these could be compared if need be - as data is normalised within each cohort this does not seem critical. Finally, antigen 11 appears to be in its own cohort (no other yellow groups) and also seems to be far below the normalised level of 1 - is this actually showing protection? Interesting also that 10 (which showed a trend for protection), and 11 (which looks like it may do also) are both members of the SmLy6 family.

I confirm that I have read this submission and believe that I have an appropriate level of expertise to confirm that it is of an acceptable scientific standard.

# Peer review: Reviewer response for version 1

**DOI:** 10.21956/wellcomeopenres.16943.r36796

**Published:** 2019-10-17

**Authors:** Peter J. Hotez

**Affiliations:** 1 Texas Children’s Hospital Center for Vaccine Development, Departments of Pediatrics and Molecular Virology and Microbiology, National School of Tropical Medicine, Baylor College of Medicine, Houston, TX, USA

There's an urgent need to optimize in silico and so-called reverse vaccinology (RV) approaches for parasite vaccines, and bring this to the same status as bacterial vaccines. For example, BEXSERO is now the first licensed bacterial vaccine developed through an RV approach. The study here is an important attempt to apply RV for schistosomiasis. While in this initial attempt no promising antigen was identified, there are opportunities to both enlarge the number of potential candidates and add an extra level of screening for downselection. For example, it would be of interest to modify the approach to include human serologic screening against putatively resistant individuals in endemic areas. This approach was used to develop Sm-TSP-2 now in clinical trials.

I confirm that I have read this submission and believe that I have an appropriate level of expertise to confirm that it is of an acceptable scientific standard.
